# The Red Blood Cell Distribution Width-to-Albumin Ratio’s Role in Parkinson’s Disease: A Cross-Sectional Study

**DOI:** 10.3390/jcm14144908

**Published:** 2025-07-10

**Authors:** Fujun Liu, Qibo Ran, Zhongyu Li, Jing Chen

**Affiliations:** 1 State Key Laboratory of Biotherapy Center, West China Hospital, Sichuan University, Chengdu 610041, China; lfj2021@scu.edu.cn; 2 Department of Ophthalmology, West China Hospital, Sichuan University, Chengdu 610041, China; ranqibo@wchscu.cn; 3 Department of Neurosurgery and Neuromodulation Center, West China Hospital, Sichuan University, Chengdu 610041, China; lizhongyu@stu.scu.edu.cn

**Keywords:** Parkinson’s disease, National Health and Nutrition Examination Survey, red blood cell distribution width-to-albumin ratio, RAR

## Abstract

**Background**: The red blood cell distribution width-to-albumin ratio (RAR) serves as an indicator of systemic inflammation and nutritional status. The precise relationship between the RAR and Parkinson’s disease (PD) prevalence remains unclear. **Methods**: This study examines the association between the RAR and PD in U.S. adults aged over 40, utilizing data from the NHANES (2003–2018). Logistic regression, subgroup analyses, and restricted cubic spline (RCS) models were utilized to evaluate the relationship between the RAR and PD prevalence. **Results:** Of 22,617 participants, 287 had PD. The mean RAR was higher in PD (3.32 ± 0.04) vs. that in non-PD (3.16 ± 0.01; *p* < 0.0001). Each unit increase in the RAR was linked to a 47% rise in the PD odds (OR = 1.47; 95% CI, 1.16–1.86; *p* < 0.05). The prevalence of PD in the highest quintile (Q3) was 1.921 times higher than that in the lowest quintile (Q1) (OR = 1.921; 95% CI, 1.128–3.270). Higher RAR values were significantly associated with increased odds of PD prevalence (*p*-values for trend < 0.05). The RCS analysis indicated a nonlinear association between the RAR and PD prevalence odds (*p* = 0.0423), with RARs ≥ 3.12 associated with increased odds of PD prevalence. Subgroup analyses and interaction tests validated the robustness of the findings regarding the association. **Conclusions**: This study found a positive nonlinear relationship between the RAR and PD prevalence. The odds of PD prevalence increased notably when the RAR exceeded approximately 3.12, and they continued to rise with increasing RARs. Due to the cross-sectional design, causality cannot be confirmed. Further research is needed to explore the mechanisms linking the RAR and PD.

## 1. Introduction

The pathogenesis of Parkinson’s disease (PD), the second most prevalent neurodegenerative disorder, is characterized by a complex interplay between inflammation and metabolic regulation [1,2,3]. Recently, the role of biomarkers based on inflammation and nutritional status in disease prediction has attracted much attention, among which the red blood cell distribution width-to-albumin ratio (RAR) has become a potential research hotspot because of its integration of information on both systemic inflammation and nutritional status [4,5].

As an indicator of erythrocyte volume heterogeneity, the red blood cell distribution width is not only used in the differential diagnosis of anemia but is also associated with various pathological conditions, such as cardiovascular disease, diabetes, and chronic inflammation [6]. Its elevation may reflect oxidative stress, chronic inflammation, or abnormal erythropoiesis [2,7]. Albumin, a major protein synthesized by the liver, is a sensitive indicator of nutritional status and also participates in the disease process by regulating the inflammatory response [8]. The RAR, by integrating these two routine parameters, is able to reflect the inflammation–nutritional imbalance of the body in a more comprehensive way. Research indicates that the RAR has independent prognostic value in diseases such as acute myocardial infarction [9], heart failure [10], and chronic kidney disease [11] and is significantly and positively associated with all-cause mortality in the general population [4].

In the field of neurodegenerative diseases, chronic inflammation and oxidative stress have been proven to be involved in PD progression [12,13]. While an elevated red blood cell distribution width is associated with cognitive impairment and dementia risk [14], its predictive value in PD is limited when used alone. A study by Kenangil et al. found that although PD patients had higher red blood cell distribution width levels than those of healthy individuals, there was no significant correlation with the disease progression [15]. However, the use of the combination of red blood cell distribution width levels with albumin to form the RAR in PD studies is limited. The study of Jia et al. identified a notable link between this indicator and PD mortality in individuals aged 20 years and above, highlighting its potential for evaluating the PD risk and prognosis [4]. Building upon these findings, we infer that the underlying mechanisms linking the RAR to PD likely involve chronic inflammation, oxidative stress, and nutritional imbalance. An elevated red blood cell distribution width, as a component of the RAR, is a well-recognized marker of inflammation and oxidative stress, both critical contributors to PD neurodegeneration. Chronic systemic inflammation can lead to the sustained activation of microglia and astrocytes, driving neuroinflammatory responses that directly harm dopaminergic neurons [16]. Concurrently, oxidative stress, indicated by an increased red blood cell distribution width, contributes to mitochondrial dysfunction and the abnormal aggregation of α-synuclein, key pathological events in PD [17]. Furthermore, albumin, the other component of the RAR, is a crucial antioxidant and transporter molecule. Decreased albumin levels reflect poor nutritional status, reduced antioxidant capacity, and increased systemic inflammation, all of which can exacerbate neuronal vulnerability and disease progression in PD [18]. Thus, the RAR, by simultaneously reflecting these interconnected pathophysiological pathways, potentially serves as a comprehensive indicator of the inflammatory and nutritional milieu contributing to PD. Nevertheless, the precise relationship between the RAR and PD prevalence remains unclear. It is necessary to ascertain whether the association is linear or nonlinear. It is also important to determine whether there is a precise threshold effect, and whether there are interactions between different subgroups.

This study aimed to elucidate the exact relationship between the RAR and the PD prevalence. Exploring the RAR in PD could reveal new connections between the RAR and neurodegenerative processes. We analyzed data from the 2003–2018 National Health and Nutrition Examination Survey (NHANES) to evaluate the precise association between the RAR and PD prevalence in U.S. adults over 40. Furthermore, while the study by Jia et al. included individuals aged 20 years and above, given that PD is exceedingly rare before age 40 and its prevalence significantly increases with age, our study specifically focused on individuals aged 40 years older. This aimed to provide more clinically pertinent insights into the primary demographic affected by PD, and to offer a more targeted analysis for the population at the highest risk. The objective of this study was not to establish causality. Instead, our study’s findings offer initial evidence to guide future longitudinal research aimed at clarifying the specific physiological mechanisms underlying the relationship between the RAR and PD. This could lead to the creation of novel biomarkers for early PD screening and risk stratification.

## 2. Materials and Methods

### 2.1. Study Design and Population

**Figure 1:**
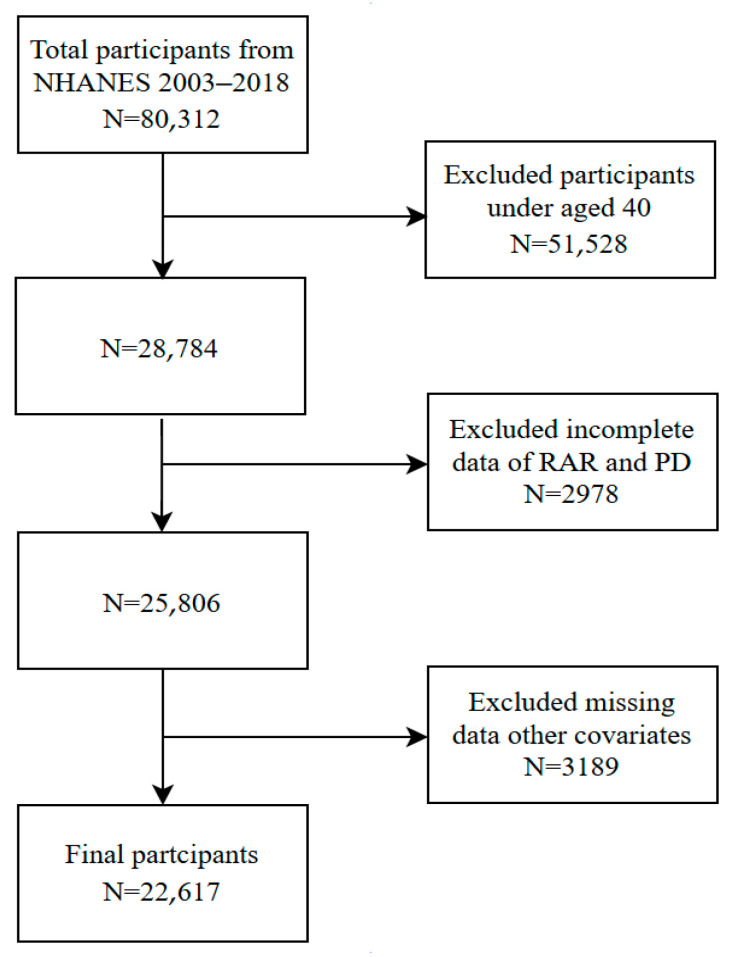
Diagram illustrating the process of selecting the study participants. PD, Parkinson’s disease; NHANES, National Health and Nutrition Examination Survey.

This study utilized publicly available data from the NHANES. We selected NHANES cycles from 2003 to 2018 because these cycles consistently collected data on PD medication use and included all the necessary laboratory measures (red blood cell distribution width and albumin) and a comprehensive set of covariates relevant to our study. This period also provided a sufficiently large sample size to ensure adequate statistical power for our analysis of a relatively rare condition like PD. The NHANES is an ongoing cross-sectional survey by the National Center for Health Statistics (NCHS) under the Centers for Disease Control and Prevention (CDC). The survey evaluates the health and nutritional status of adults and children in the U.S. The survey uses a stratified, multi-stage probability sampling method to obtain a nationally representative sample of the non-institutionalized U.S. population. The data collection process includes household interviews, standardized physical examinations conducted in mobile examination centers, and laboratory tests. All participants in the NHANES study gave written informed consent, and the NCHS Research Ethics Review Board approved the study protocols. This secondary analysis of publicly available de-identified data was deemed exempt from additional Institutional Review Board approval. The NHANES data can be downloaded for free from the CDC website at https://www.cdc.gov/nchs/nhanes/ (accessed on 16 May 2025). From a total of 80,312 participants, we excluded those under 40 years, those with incomplete data for the RAR and PD, and those with missing data for other covariates. Following the conclusion of the present study, a total of 22,617 participants were included in the final analysis (Figure 1).

### 2.2. Exposure and Outcome Definitions

In this study, the exposure variable was defined as the RAR. The RAR was calculated as the ratio of the red blood cell distribution width to serum albumin (g/dL). Peripheral blood samples were analyzed for their red blood cell distribution widths using a Coulter analyzer at the mobile examination centers. The serum albumin concentration (g/dL) was measured using the bromocresol purple method. All laboratory tests were conducted following standardized protocols to ensure data validity and comparability. The presence of a PD diagnosis was established as the outcome. PD was defined based on the participants’ self-reported use of specific anti-PD medications, as ascertained through the prescription medication questionnaire in the NHANES. Participants were identified as having PD if they reported using any of the following medications: carbidopa, entacapone, levodopa, amantadine, pramipexole, or bromocriptine [12]. Participants who did not report using these medications were classified as non-PD individuals. This method for identifying PD cases based on anti-PD medication use in the NHANES database is consistent with previous studies and has been shown to be a valid approach given the available data limitations [12,19].

### 2.3. Covariates

Potential confounders were selected based on the existing literature and their clinical relevance. The demographic variables comprised age, gender, ethnicity (categorized White, Black, Hispanic, Mexican American, or other), education level (less than high school, high school graduate, or college and above), marital status (married or unmarried), and poverty-to-income ratio (PIR), along with smoking status (nonactive or active smoker). Clinical measurements included the body mass index (BMI). Chronic comorbidities, including hypertension, diabetes mellitus (DM), hyperlipidemia, coronary heart disease, chronic kidney disease (CKD), and stroke, were identified based on self-reported physician diagnoses, medication usage, or clinical criteria. Laboratory parameters included the white blood cell, alanine aminotransferase (ALT), aspartate aminotransferase (AST), globulin, uric acid, sodium, phosphorus, and total calcium levels.

### 2.4. Statistical Analysis

The statistical analyses were performed using R software (version 4.2.2). Descriptive statistics were calculated to summarize the characteristics of the study participants. Continuous variables are expressed as weighted means with standard deviations, while categorical variables are shown as frequencies and percentages. Weighted *t*-tests and chi-square tests were used to compare the baseline characteristics between the groups for the continuous and categorical variables, respectively. The association between the RAR and the odds of PD was assessed using weighted logistic regression models. The RAR was examined as both a continuous and quartile-based categorical variable. A series of multi-variable models were constructed: a crude model (unadjusted) and adjusted models controlling for potential confounders in a stepwise manner (corresponding to different adjustment levels). To investigate the potential nonlinear relationship between the continuous RAR and the odds of PD, a restricted cubic spline (RCS) analysis was conducted, adjusting for all variables. We used 3 knots (chosen based on the Akaike Information Criterion (AIC) and placed at the 10th, 50th, and 90th percentiles of the RAR distribution) to model the nonlinear relationship. The analysis was performed using the “rms” package (version 6.8-2) in R software. The threshold value (3.12) was determined as the point where the slope of the spline curve showed a statistically significant change, indicating an accelerated increase in the odds of PD prevalence beyond this point. Subgroup analyses assessed the consistency of the association across different stratifying factors, such as age, gender, marital status, stroke, PIR, BMI, hyperlipidemia, DM, hypertension, coronary heart disease, smoking status, and CKD. Interaction terms between the RAR and each stratifying variable were incorporated into the models to formally test for effect modification. Statistical significance was defined as a two-sided *p*-value < 0.05.

## 3. Results

### 3.1. Characteristics of Participants

The comprehensive characterization of the 22,617 eligible participants is shown in Table 1. Among the participants, there were 287 individuals with PD and 22,330 without, and 52.57% were women and 47.43% were men. The two groups exhibited notable differences in the various demographic and clinical variables. Participants with PD were older, and differences were noted in their gender distribution and ethnicity. The socioeconomic status, as indicated by the PIR, also differed significantly. The prevalence of several comorbidities was significantly higher in the participants with PD, including stroke (13.59% vs. 4.22%, *p* < 0.0001), DM (20.23% vs. 14.38%, *p* = 0.02), CKD (36.98% vs. 19.51%, *p* < 0.0001), coronary heart disease (9.01% vs. 5.44%, *p* = 0.03), and hypertension (98.42% vs. 95.87%, *p* = 0.03). Among the laboratory parameters, the RAR (3.32 ± 0.04 vs. 3.16 ± 0.01, *p* < 0.0001), WBC (7.57 ± 0.17 vs. 7.16 ± 0.03, *p* = 0.02), and creatinine (0.97 ± 0.02 vs. 0.92 ± 0.00, *p* = 0.04) showed significant differences between the groups. There were no significant differences in terms of marital status, education level, BMI, smoking status, hyperlipidemia, ALT, AST, globulin, uric acid, sodium, phosphorus, or total calcium.

### 3.2. Association of RAR and PD

Table 2 presents the findings of the unadjusted logistic regression models regarding the odds of PD prevalence. Several factors were significantly associated with increased odds of PD. Age ≥ 65 years (OR = 1.65, 95% CI: 1.22–2.22, *p* = 0.001), female gender (OR = 1.44, 95% CI: 1.04–1.98, *p* = 0.03), CKD (OR = 2.42, 95% CI: 1.76–3.34, *p* < 0.0001), diabetes mellitus (OR = 1.51, 95% CI: 1.07–2.13, *p* = 0.02), coronary heart disease (OR = 1.72, 95% CI: 1.04–2.85, *p* = 0.03), hypertension (OR = 2.68, 95% CI: 1.05–6.84, *p* = 0.04), stroke (OR = 3.56, 95% CI: 2.38–5.34, *p* < 0.0001), an elevated RAR (OR = 1.71, 95% CI: 1.43–2.06, *p* < 0.0001), and higher creatinine levels (OR = 1.17, 95% CI: 1.07–1.28, *p* < 0.001) were associated with increased odds of PD. Conversely, a PIR ≥ 2, Black ethnicity, and other ethnicity showed decreased odds of PD (all *p* < 0.05). Other variables were not significantly associated with PD. Table 3 presents the association between the RAR and the odds of PD across the different multivariate logistic regression models. In the crude model, the continuous RAR was significantly associated with increased odds of PD (OR = 1.71, 95% CI: 1.43–2.06, *p* < 0.0001). This significant association persisted in Model 1, Model 2, and notably, Model 3 (OR = 1.47, 95% CI: 1.16–1.86, *p* = 0.001), which adjusted for a comprehensive set of covariates, including age, sex, and various comorbidities. This demonstrates that the RAR is independently associated with PD prevalence, even after accounting for the influence of other known factors. When the RAR was analyzed by quartiles, compared to the lowest quartile (Q1, reference), Q2 showed significantly increased odds of PD in the crude model (OR = 1.65, 95% CI: 1.09–2.50, *p* = 0.018) and Model 1 (OR = 1.56, 95% CI: 1.02–2.36, *p* = 0.039), but this association became marginally non-significant in Model 2 (OR = 1.49, 95% CI: 0.99–2.27, *p* = 0.059) and Model 3 (OR = 1.48, 95% CI: 0.96–2.30, *p* = 0.076). Q3 consistently showed significantly increased odds of PD across all models: crude (OR = 2.40, 95% CI: 1.52–3.79, *p* < 0.001), Model 1 (OR = 2.184, 95% CI: 1.32–3.60, *p* = 0.002), Model 2 (OR = 1.95, 95% CI: 1.16–3.29, *p* = 0.013), and Model 3 (OR = 1.92, 95% CI: 1.13–3.27, *p* = 0.017). The trends of the *p*-values across the RAR quartiles were significant in all the models (crude: *p* < 0.001; Model 1: *p* = 0.002; Model 2: *p* = 0.013; Model 3: *p* = 0.016), indicating a dose–response relationship between an increasing RAR and the odds of PD.

### 3.3. Evaluation of Nonlinear Relationship Between RAR and PD

**Figure 2:**
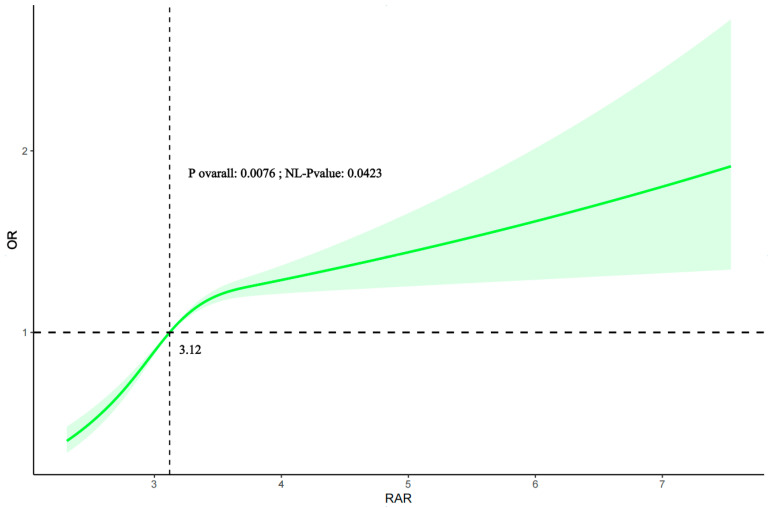
Shows the weighted association between the RAR and PD incidence using the restricted cubic spline model, adjusted for all covariates in Model 3. The solid green line indicates a smooth curve fit between the variables, while the green line indicates the 95% confidence interval.

An RCS model was employed to investigate the potential linear or nonlinear relationship between the RAR and PD based on Model 3 (Figure 2). The results indicated that the overall *p*-value for the association was 0.0076, and the *p*-value for nonlinearity was 0.0423, suggesting a significant nonlinear relationship between the RAR and the odds of PD. Specifically, the odds of PD starts to increase notably when the RAR exceeds approximately 3.12, and it continues to rise with the increasing RAR, indicating a dose–response pattern beyond this threshold.

### 3.4. Subgroup Analysis

**Figure 3:**
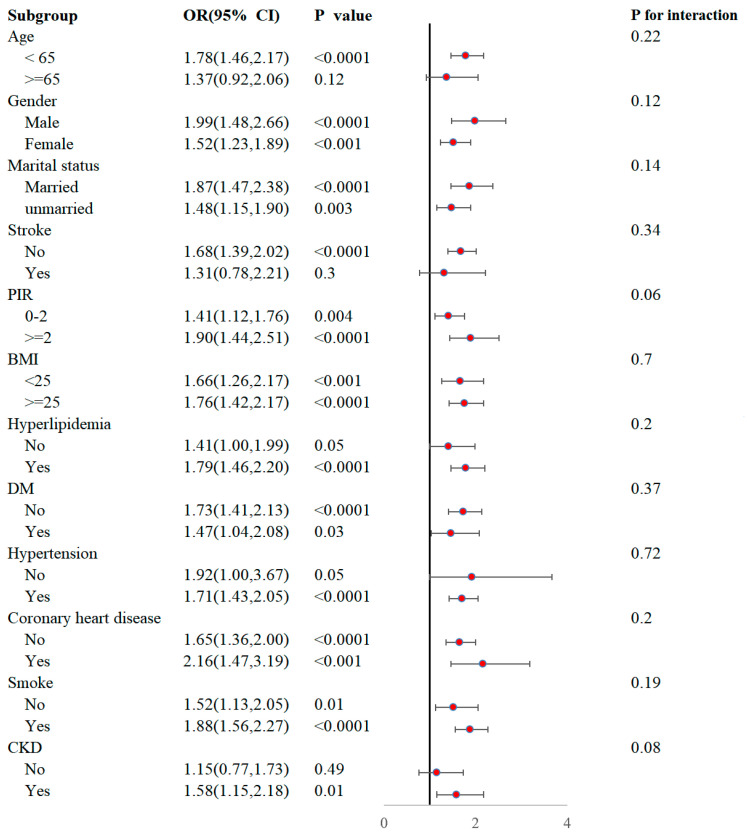
Subgroup analysis of the relationship between the RAR and PD. No statistical differences between subgroups were observable from the graphs (all *p*-values for interactions ≥ 0.05). BMI, body mass index; CI, confidence interval; CKD, chronic kidney disease; DM, diabetes mellitus; OR, odds ratio; PIR, poverty-to-income ratio; PD, Parkinson’s disease; RAR, red blood distribution width-to-albumin ratio.

We stratified the categorical variables among the covariates to analyze the association between the RAR and the odds of PD prevalence across different subgroups. These covariates included age, gender, marital status, history of stroke, PIR, BMI, hyperlipidemia, DM, hypertension, coronary heart disease, smoking status, and CKD. The RAR demonstrated a significant association with increased odds of PD in most subgroups. For instance, significant associations were observed in individuals aged < 65 years (OR = 1.78, 95% CI: 1.46–2.17), males (OR = 1.99, 95% CI: 1.48–2.66), married individuals (OR = 1.87, 95% CI: 1.47–2.38), those with PIRs ≥ 2 (OR = 1.90, 95% CI: 1.44–2.51), those with BMIs ≥ 25 (OR = 1.76, 95% CI: 1.42–2.17), individuals with hyperlipidemia (OR = 1.79, 95% CI: 1.46–2.20), individuals with hypertension (OR = 1.71, 95% CI: 1.43–2.05), individuals with coronary heart disease (OR = 2.16, 95% CI: 1.47–3.19), current smokers (OR = 1.88, 95% CI: 1.56–2.27), and those with CKD (OR = 1.58, 95% CI: 1.15–2.18). Importantly, the formal tests for interaction revealed no statistically significant differences in the association between the RAR and PD across any of the evaluated subgroups (all *p*-values for interaction > 0.05) (Figure 3).

## 4. Discussion

This large cross-sectional study, utilizing NHANES data from 2003 to 2018, identified a significant positive association between the RAR and the prevalence of PD in U.S. adults aged 40 and older. Notably, the association between the RAR and PD remained statistically significant even after extensive adjustments for potential confounders, including age, suggesting an independent association between the RAR and PD prevalence. Furthermore, the RCS analysis demonstrated a nonlinear association between the RAR and the likelihood of PD prevalence. We identified a threshold of approximately 3.12 for the RAR, above which the odds of PD prevalence significantly increased and continued to rise with the increasing RAR. To the best of our knowledge, this is the first study to identify such a nonlinear association and specific threshold for the RAR in relation to PD prevalence. Subgroup analyses confirmed the robustness of this association across various demographic and clinical factors. These results highlight the RAR as a potential indicator of the inflammatory–nutritional status associated with PD prevalence, suggesting that a threshold-dependent mechanism might be involved.

Numerous studies have indicated that the RAR is an emerging biomarker that is closely associated with the onset, progression, and prognoses of a variety of diseases. In a study of depression, Zhou et al. found a significant positive correlation between the RAR and the prevalence of depression [20]. Further analyses revealed a linear association between the RAR and depression, with a significant increase in depression when the RAR exceeded 3.16, which continued to rise with higher RAR values [20]. Eyiol et al. found that the RAR serves as a feasible index for prognosis and severity stratification in acute ischemic stroke [21]. Furthermore, Hao et al. demonstrated that higher RAR levels were linked to a greater risk of both all-cause and cause-specific mortalities in the general population [5]. Collectively, these studies highlight the significant clinical value of the RAR as a prognostic biomarker and risk stratification tool.

Recent research has been increasingly focused on the impact of systemic inflammation and nutritional status in the progression and development of PD [22,23]. Qin et al. investigated the red cell distribution width in 94 PD patients and 279 healthy controls, demonstrating that the PD patients had significantly elevated red blood cell distribution width levels compared to those of the healthy controls, and that the red blood cell distribution width was positively correlated with PD motor scores [24]. Shen et al. reported that albumin levels could predict cognitive decline in individuals with PD [25]. However, research combining the red blood cell distribution width and albumin as combined biomarkers in PD has been limited. The integration of the red blood cell distribution width and albumin into a single ratio (RAR) provides a more comprehensive and potentially more meaningful indicator than the red blood cell distribution width alone [21]. While the red blood cell distribution width primarily reflects the inflammatory and oxidative stress states, albumin, as a critical plasma protein, is involved in maintaining oncotic pressure, transporting various substances, and acting as an important antioxidant [4]. Low albumin levels often signify malnutrition, systemic inflammation, or liver dysfunction. Therefore, the RAR simultaneously captures information regarding both inflammatory processes (from the red blood cell distribution width) and nutritional/antioxidant status (from albumin). Since both chronic inflammation and malnutrition are prevalent in PD patients and are increasingly recognized as contributing factors to its pathogenesis and progression, the RAR offers a more integrated and holistic biomarker that reflects the complex interplay of these factors [25]. This combined measure may thus provide a more robust and sensitive assessment of systemic pathological states relevant to PD than either component alone. In a study of PD, Jia et al. found that the RAR is significantly associated with PD mortality, underscoring its potential in assessing the PD risk and prognosis [4]. However, there are notable distinctions between our research and previous studies. Our study uniquely quantifies the relationship between the RAR and the odds of PD prevalence, an association not previously explored in the existing research. This study elucidates the nonlinear correlation between the RAR and PD prevalence, identifying a threshold around 3.12 for the RAR, beyond which the likelihood of PD prevalence notably increases and continues to escalate with higher RAR values.

The observed nonlinear positive correlation between the RAR and the prevalence of PD may be attributed to a confluence of underlying mechanisms, primarily involving chronic inflammation, oxidative stress, and nutritional imbalance/metabolic dysfunction. Firstly, an elevated RAR may reflect a state of persistent chronic inflammation [2]. An increased red blood cell distribution width is associated with elevated levels of inflammatory cytokines such as interleukin-6 and TNF-α, while low albumin levels are commonly observed during inflammatory responses [2,5]. Chronic inflammation can activate microglia, promoting neuroinflammatory responses that lead to the damage of dopaminergic neurons, thereby increasing the PD risk [26]. Secondly, oxidative stress injury plays a significant role. An increased red blood cell distribution width is linked to increased levels of oxidative stress markers, such as reactive oxygen species [2]. Oxidative stress contributes to mitochondrial dysfunction and the abnormal aggregation of α-synuclein, key pathological hallmarks of PD [2,4]. Furthermore, low albumin levels may compromise its antioxidant capacity, exacerbating neuronal damage [5]. Lastly, nutritional imbalance and metabolic abnormalities are implicated. Decreased albumin levels often indicate malnutrition or metabolic disturbances secondary to chronic disease. Nutritional deficiencies can impair the neuronal energy supply and repair mechanisms. An elevated RAR is linked to metabolic disorders, including diabetes and obesity [27,28], which indirectly contribute to PD development via insulin resistance and abnormal glucose metabolism [29,30]. Collectively, these interconnected pathways involving chronic inflammation, oxidative stress, and metabolic dysregulation likely contribute to the nonlinear relationship between the RAR and the increased odds of PD.

The strengths of this study can be highlighted in several key areas. First, we employed a nationally representative and sufficiently large sample from the U.S. population, enhancing the generalizability of our results. Second, to ensure the reliability of the findings, we utilized appropriate statistical weights and adjusted for both known and potential confounders that may influence the relationship between the RAR and PD. Lastly, we applied the RCS and smooth-curve-fitting method to explore the nonlinear relationship between the RAR and the odds of PD. However, this research has several limitations. Firstly, although we considered various factors, our results may still be influenced by measurement bias and unmeasured confounding factors. The cross-sectional design limits our ability to infer causality; thus, while we documented a significant association between the RAR and PD prevalence, we cannot establish a causal relationship. Future longitudinal studies, such as those employing Mendelian randomization, are needed to explore the causal pathways between the RAR and PD more definitively. Additionally, PD diagnosis was based on self-reported medication use, which may have led to misclassification, as some individuals in the NHANES may have been unaware of their PD symptoms. This could introduce bias into our findings. Fourthly, our identification of PD patients relied on the self-reported use of anti-PD medications, including bromocriptine. However, bromocriptine is currently rarely used for PD treatment and is more commonly prescribed for other conditions, such as hyperprolactinemia and acromegaly. This could have led to the misclassification of the PD status, potentially underestimating the true prevalence of PD in our study. Our study also relied on a single baseline measurement of the RAR, which does not capture the dynamic nature of this biomarker over time. Variability in the RAR levels throughout the study period may be associated with PD prevalence more accurately than a single measurement can reflect. The longitudinal tracking of the RAR and its fluctuations would provide a more comprehensive understanding of its temporal dynamics and their impact on PD prevalence. Moreover, this research focused exclusively on U.S. adults over 40 with PD, limiting the generalizability of our findings to other populations. Future research should incorporate diverse and international cohorts to validate the clinical utility of the RAR across different demographic groups.

## 5. Conclusions

This study examined a large cross-sectional population of U.S. adults with PD aged over 40 years, utilizing NHANES data from 2003 to 2018. Our major finding indicates a significant positive association between the RAR and the prevalence of PD. Notably, we discovered a threshold of approximately 3.12 for the RAR, above which the odds of PD prevalence significantly increased, suggesting a nonlinear relationship. This research is the first to establish such a threshold and highlight the nonlinear association between the RAR and PD, underscoring the RAR as a potential biomarker for the inflammatory–nutritional status linked to PD prevalence. Additional research is needed to clarify the physiological mechanisms linking the RAR and PD, and to validate these findings through prospective studies.

## Figures and Tables

**Table 1:** Weighted characteristics of the study participants in NHANES 2003–2018.

Variable	Total	Non-Parkinson	Parkinson	*p*-Value
No.	22,617	22,330	287	
Age (years), mean + SD	57.91 ± 0.16	57.87 ± 0.16	61.52 ± 1.04	<0.001
Gender, n (%)				0.03
Male	11,117 (47.43)	10,978 (47.54)	139 (38.64)	
Female	11,500 (52.57)	11,352 (52.46)	148 (61.36)	
Marital status, n (%)				0.14
Married	14,070 (67.98)	13,911 (68.04)	159 (62.78)	
Unmarried	8547 (32.02)	8419 (31.96)	128 (37.22)	
Education level, n (%)				0.57
Less than high school	2993 (6.28)	2956 (6.28)	37 (6.82)	
High school	8376 (34.16)	8262 (34.12)	114 (37.08)	
More than high school	11,248 (59.56)	11,112 (59.60)	136 (56.10)	
Ethnicity, n (%)				0.003
White	10,699 (74.11)	10,512 (74.01)	187 (82.44)	
Black	4652 (9.64)	4613 (9.67)	39 (7.31)	
Mexican American	3243 (5.72)	3213 (5.73)	30 (4.49)	
Other	4023 (10.53)	3992 (10.59)	31 (5.75)	
BMI				0.74
25	5738 (25.89)	5670 (25.87)	68 (26.98)	
≥25	16,879 (74.11)	16,660 (74.13)	219 (73.02)	
PIR				<0.001
0–2	10,183 (29.75)	10,016 (29.60)	167 (42.48)	
≥2	12,434 (70.25)	12,314 (70.40)	120 (57.52)	
Smokes				0.8
No	11,461 (51.26)	11,324 (51.25)	137 (52.14)	
Yes	11,156 (48.74)	11,006 (48.75)	150 (47.86)	
Stroke				<0.0001
No	21,319 (95.66)	21,072 (95.78)	247 (86.41)	
Yes	1298 (4.34)	1258 (4.22)	40 (13.59)	
Hyperlipidemia				0.49
No	4512 (19.62)	4464 (19.65)	48 (17.16)	
Yes	18,105 (80.38)	17,866 (80.35)	239 (82.84)	
DM				0.02
No	18,279 (85.55)	18,069 (85.62)	210 (79.77)	
Yes	4338 (14.45)	4261 (14.38)	77 (20.23)	
CKD				<0.0001
No	16,813 (80.28)	16,644 (80.49)	169 (63.02)	
Yes	5804 (19.72)	5686 (19.51)	118 (36.98)	
Coronary heart disease				0.03
No	21,175 (94.52)	20,921 (94.56)	254 (90.99)	
Yes	1442 (5.48)	1409 (5.44)	33 (9.01)	
Hypertension				0.03
No	925 (4.10)	919 (4.13)	6 (1.58)	
Yes	21,692 (95.90)	21,411 (95.87)	281 (98.42)	
RAR	3.16 ± 0.01	3.16 ± 0.01	3.32 ± 0.04	<0.0001
WBC	7.17 ± 0.03	7.16 ± 0.03	7.57 ± 0.17	0.02
ALT_ IU/L	25.11 ± 0.15	25.12 ± 0.15	24.11 ± 1.64	0.54
AST_ IU/L	25.71 ± 0.13	25.70 ± 0.13	26.63 ± 1.64	0.57
Globulin_g.dL	2.83 ± 0.01	2.83 ± 0.01	2.82 ± 0.04	0.73
Creatinine_mg.dL	0.92 ± 0.00	0.92 ± 0.00	0.97 ± 0.02	0.04
Uric acid_umol.L	326.57 ± 0.85	326.52 ± 0.85	331.20 ± 6.53	0.47
Sodium_mmol.L	139.35 ± 0.07	139.35 ± 0.07	139.20 ± 0.19	0.36
Phosphorus_mmol.L	1.20 ± 0.00	1.20 ± 0.00	1.21 ± 0.02	0.75
Calcium total_mg.dL	9.42 ± 0.01	9.42 ± 0.01	9.37 ± 0.03	0.15

AST, aspartate aminotransferase; ALT, alanine aminotransferase; BMI, body mass index; CKD, chronic kidney disease; DM, diabetes mellitus; No., number; PIR, poverty-to-income ratio; RAR, red blood distribution width-to-albumin ratio; WBC, white blood cell.

**Table 2:** Unadjusted logistic regression models of PD.

Variables	OR (95% CI)	*p*-Value
Age		
<65	Ref.	Ref.
≥65	1.65 (1.22, 2.22)	0.001
Gender, n (%)		
Male	Ref.	Ref.
Female	1.44 (1.04, 1.98)	0.03
Marital status, n (%)		
Married	Ref.	Ref.
Unmarried	1.26 (0.92, 1.73)	0.14
PIR		
0–2	Ref.	Ref.
≥2	0.57 (0.41, 0.78)	<0.001
Education level, n (%)		
Less than high school	Ref.	Ref.
High school	1.00 (0.62, 1.61)	1
More than high school	0.87 (0.58, 1.29)	0.47
Ethnicity, n (%)		
White	Ref.	Ref.
Black	0.68 (0.47, 0.97)	0.04
Mexican American	0.70 (0.48, 1.02)	0.06
Other	0.49 (0.29, 0.83)	0.01
BMI		
<25	Ref.	Ref.
≥25	0.94 (0.67, 1.33)	0.74
Smokes		
No	Ref.	Ref.
Yes	0.96 (0.73, 1.28)	0.8
Hyperlipidemia		
No	Ref.	Ref.
Yes	1.18 (0.73, 1.91)	0.49
CKD		
No	Ref.	Ref.
Yes	2.42 (1.76, 3.34)	<0.0001
DM		
No	Ref.	Ref.
Yes	1.51 (1.07, 2.13)	0.02
Coronary heart disease		
No	Ref.	Ref.
Yes	1.72 (1.04, 2.85)	0.03
Hypertension		
No	Ref.	ref
Yes	2.68 (1.05, 6.84)	0.04
Stroke		
No	Ref.	Ref.
Yes	3.56 (2.38, 5.34)	<0.0001
RAR	1.71 (1.43, 2.06)	<0.0001
WBC	1.01 (1.00, 1.03)	0.1
ALT_ IU/L	1.00 (0.98, 1.01)	0.63
AST_ IU/L	1.00 (1.00, 1.01)	0.31
Globulin_g.dL	0.94 (0.66, 1.34)	0.73
Creatinine_mg.dL	1.17 (1.07, 1.28)	<0.001
Uric acid_umol.L	1.00 (1.00, 1.00)	0.47
Sodium_mmol.L	0.97 (0.92, 1.03)	0.35
Phosphorus_mmol.L	1.17 (0.43, 3.17)	0.75
Calcium total_mg.dL	0.70 (0.42, 1.15)	0.16

AST, aspartate aminotransferase; ALT, alanine aminotransferase; BMI, body mass index; CKD, chronic kidney disease; DM, diabetes mellitus; OR, odds ratio; PIR, poverty-to-income ratio; RAR, red blood distribution width-to-albumin ratio; Ref., reference; WBC, white blood cell.

**Table 3:** The association between the RAR and PD prevalence by multivariate logistical models.

	Crude Model*		Model 1*		Model 2*		Model 3*	
Character	95%CI	*p*	95%CI	*p*	95%CI	*p*	95%CI	*p*
Continuous	1.71 (1.43, 2.06)	<0.0001	1.65 (1.32, 2.07)	<0.0001	1.50 (1.18, 1.91)	0.001	1.47 (1.16, 1.86)	0.001
RAR quartiles								
Q1	Ref.		Ref.		Ref.		Ref.	
Q2	1.65 (1.09, 2.50)	0.018	1.56 (1.02, 2.36)	0.039	1.49 (0.99, 2.27)	0.059	1.48 (0.96, 2.30)	0.076
Q3	2.40 (1.52, 3.79)	<0.001	2.184 (1.32, 3.60)	0.002	1.95 (1.16, 3.29)	0.013	1.92 (1.13, 3.27)	0.017
*p* for trend		<0.001		0.002		0.013		0.016

The crude model* was devoid of covariates. Model 1* contained only age, education, sex, ethnicity, marital status, and poverty-to-income ratio. Model 2* further included diabetes, hyperlipidemia, stroke, hypertension, coronary heart disease, smoking status, body mass index, and chronic kidney disease. Model 3* incorporated all covariates (age, education, sex, ethnicity, marital status, poverty-to-income ratio, diabetes, hyperlipidemia, stroke, hypertension, coronary heart disease, smoking status, body mass index, chronic kidney disease, white blood cell counts, alanine and aspartate aminotransferase levels, creatinine, phosphorus, globulin, uric acid, sodium, and calcium).

## Data Availability

The datasets used in this study are available in online repositories. The repository names and their accession numbers are available at https://www.cdc.gov/nchs/nhanes/ (accessed on 16 May 2025). If you have any further questions, please reach out to the corresponding author directly.

## Abbreviations

The following abbreviations are used in this manuscript:

ALT	Alanine aminotransferase
AST	Aspartate aminotransferase
BMI	Body mass index
CI	Confidence interval
CKD	Chronic kidney disease
DM	Diabetes mellitus
NHANES	National Health and Nutrition Examination Survey
OR	Odds ratio
PD	Parkinson’s disease
PIR	Poverty-to-income ratio
RAR	Red blood distribution width-to-albumin ratio

## References

[1] Kwok J.Y.Y., Chan L.M.L., Lai C.A., Ho P.W.L., Choi Z.Y., Auyeung M., Pang S.Y.Y., Choi E.P.H., Fong D.Y.T., Yu D.S.F. (2025). Effects of Meditation and Yoga on Anxiety, Depression and Chronic Inflammation in Patients with Parkinson’s Disease: A Randomized Clinical Trial. Psychother. Psychosom..

[2] Salvagno G.L., Sanchis-Gomar F., Picanza A., Lippi G. (2015). Red Blood Cell Distribution Width: A Simple Parameter with Multiple Clinical Applications. Crit. Rev. Clin. Lab. Sci..

[3] Carrozzino D., Siri C., Bech P. (2019). The Prevalence of Psychological Distress in Parkinson’s Disease Patients: The Brief Symptom Inventory (BSI-18) versus the Hopkins Symptom Checklist (SCL-90-R). Prog. Neuro-Psychopharmacol. Biol. Psychiatry.

[4] Jia H., Yin K., Zhao J., Che F. (2024). Association of Inflammation/Nutrition-Based Indicators with Parkinson’s Disease and Mortality. Front. Nutr..

[5] Hao M., Jiang S., Tang J., Li X., Wang S., Li Y., Wu J., Hu Z., Zhang H. (2024). Ratio of Red Blood Cell Distribution Width to Albumin Level and Risk of Mortality. JAMA Netw. Open.

[6] Haenggi E., Kaegi-Braun N., Wunderle C., Tribolet P., Mueller B., Stanga Z., Schuetz P. (2024). Red Blood Cell Distribution Width (RDW)—A New Nutritional Biomarker to Assess Nutritional Risk and Response to Nutritional Therapy?. Clin. Nutr..

[7] Moriarty P.M., Steg P.G., McGinniss J., Zeiher A.M., White H.D., Manvelian G., Pordy R., Loy M., Jukema J.W., Harrington R.A. (2022). Relation of Red Blood Cell Distribution Width to Risk of Major Adverse Cardiovascular Events, Death, and Effect of Alirocumab after Acute Coronary Syndromes. J. Clin. Lipidol..

[8] Feng K.Y., Ambrosy A.P., Zhou Z., Li D., Kong J., Zaroff J.G., Mishell J.M., Ku I.A., Scotti A., Coisne A. (2023). Association between Serum Albumin and Outcomes in Heart Failure and Secondary Mitral Regurgitation: The COAPT Trial. Eur. J. Heart Fail..

[9] Li D., Ruan Z., Wu B. (2022). Association of Red Blood Cell Distribution Width-Albumin Ratio for Acute Myocardial Infarction Patients with Mortality: A Retrospective Cohort Study. Clin. Appl. Thromb. Hemost..

[10] Ni Q., Wang X., Wang J., Chen P. (2022). The Red Blood Cell Distribution Width-Albumin Ratio: A Promising Predictor of Mortality in Heart Failure Patients—A Cohort Study. Clin. Chim. Acta.

[11] Kimura H., Tanaka K., Saito H., Iwasaki T., Kazama S., Shimabukuro M., Asahi K., Watanabe T., Kazama J.J. (2023). Impact of Red Blood Cell Distribution Width-Albumin Ratio on Prognosis of Patients with CKD. Sci. Rep..

[12] Liu F., Ran Q., Zhang H., Chen J. (2025). The Systemic Immune-Inflammation Index and the Risk of Parkinson’s Disease in the U.S.: A Cross-Sectional Study. J. Clin. Med..

[13] Weuve J., Mendes de Leon C.F., Bennett D.A., Dong X., Evans D.A. (2014). The Red Cell Distribution Width and Anemia in Association with Prevalent Dementia. Alzheimer Dis. Assoc. Disord..

[14] Beydoun M.A., Hossain S., Beydoun H.A., Shaked D., Weiss J., Evans M.K., Zonderman A.B. (2020). Red Cell Distribution Width Is Directly Associated with Poor Cognitive Performance among Nonanemic, Middle-Aged, Urban Adults. J. Nutr..

[15] Kenangil G., Ari B.C., Kaya F.A., Demir M., Domac F.M. (2020). Red Cell Distribution Width Levels in Parkinson’s Disease Patients. Acta Neurol. Belg..

[16] Wang N., Xiao X., Chen Z., Xu K., Cao X., Kou D., Zeng J. (2025). Glial Cell Crosstalk in Parkinson’s Disease: Mechanisms, Implications, and Therapeutic Strategies. Fundam. Res..

[17] Chang K.-H., Chen C.-M. (2020). The Role of Oxidative Stress in Parkinson’s Disease. Antioxidants.

[18] He H., Xiong X., Zheng Y., Hou J., Jiang T., Quan W., Huang J., Xu J., Chen K., Qian J. (2025). Parkin Characteristics and Blood Biomarkers of Parkinson’s Disease in WPBLC Study. Front. Aging Neurosci..

[19] Ke L., Zhao L., Xing W., Tang Q. (2024). Association between Parkinson’s Disease and Cardiovascular Disease Mortality: A Prospective Population-Based Study from NHANES. Lipids Health Dis..

[20] Zhou Y., Zhao L., Tang Y., Qian S. (2025). Association between Red Blood Cell Distribution Width-to-Albumin Ratio and Depression: A Cross-Sectional Analysis among US Adults, 2011–2018. BMC Psychiatry.

[21] Eyiol A., Ertekin B. (2024). Association of Red Blood Cell Distribution Width to Albumin Ratio with Prognosis in Stroke Patients. Biomark. Med..

[22] Pajares M., Rojo A.I., Manda G., Boscá L., Cuadrado A. (2020). Inflammation in Parkinson’s Disease: Mechanisms and Therapeutic Implications. Cells.

[23] Sun S., Wen Y., Li Y. (2022). Serum Albumin, Cognitive Function, Motor Impairment, and Survival Prognosis in Parkinson Disease. Medicine.

[24] Huang Z., Han Y., Hu H., Cao C., Liu D., Wang Z. (2023). Triglyceride to High-Density Lipoprotein Cholesterol Ratio Is Associated with Regression to Normoglycemia from Prediabetes in Adults: A 5-Year Cohort Study in China. J. Transl. Med..

[25] Shen J., Amari N., Zack R., Skrinak R.T., Unger T.L., Posavi M., Tropea T.F., Xie S.X., Van Deerlin V.M., Dewey R.B. (2022). Plasma MIA, CRP, and Albumin Predict Cognitive Decline in Parkinson’s Disease. Ann. Neurol..

[26] Semba R.D., Patel K.V., Ferrucci L., Sun K., Roy C.N., Guralnik J.M., Fried L.P. (2010). Serum Antioxidants and Inflammation Predict Red Cell Distribution Width in Older Women: The Women’s Health and Aging Study I. Clin. Nutr..

[27] Li D., Long J., Zhang J., He M., Zeng Q., He Q., Zhan W., Chi Y., Zou M. (2024). Association between Red Cell Distribution Width–and–Albumin Ratio and the Risk of Peripheral Artery Disease in Patients with Diabetes. Front. Endocrinol..

[28] Yu M., Pei L., Liu H., Wang J., Wen Y., Yang X., Ma C., Zhang X., Wu L., Wang L. (2024). A Novel Inflammatory Marker: Relationship Between Red Cell Distribution Width/Albumin Ratio and Vascular Complications in Patients with Type 2 Diabetes Mellitus. J. Inflamm. Res..

[29] Bousquet M., St-Amour I., Vandal M., Julien P., Cicchetti F., Calon F. (2012). High-Fat Diet Exacerbates MPTP-Induced Dopaminergic Degeneration in Mice. Neurobiol. Dis..

[30] Bachmann C.G., Zapf A., Brunner E., Trenkwalder C. (2009). Dopaminergic Treatment Is Associated with Decreased Body Weight in Patients with Parkinson’s Disease and Dyskinesias. Eur. J. Neurol..

